# Reproducible detection of disease-associated markers from gene expression data

**DOI:** 10.1186/s12920-016-0214-5

**Published:** 2016-08-18

**Authors:** Katsuhiro Omae, Osamu Komori, Shinto Eguchi

**Affiliations:** 1 Department of Statistical Science, The Graduate University for Advanced Studies, 10-3 Midori-cho, Tachikawa, Tokyo, 190–8562 Japan; 2 Department of Electrical, Electronic and Computer Engineering, University of Fukui, 3-9-1 Bunkyo, Fukui, Fukui, 910–8507 Japan; 3 The Institute of Statistical Mathematics, 10-3 Midori-cho, Tachikawa, Tokyo, 190–8562 Japan

**Keywords:** Gene expression analysis, Genes screening, Heterogeneity, Subsampling method, Two-sample test, U-statistic

## Abstract

**Background:**

Detection of disease-associated markers plays a crucial role in gene screening for biological studies. Two-sample test statistics, such as the *t*-statistic, are widely used to rank genes based on gene expression data. However, the resultant gene ranking is often not reproducible among different data sets. Such irreproducibility may be caused by disease heterogeneity.

**Results:**

When we divided data into two subsets, we found that the signs of the two *t*-statistics were often reversed. Focusing on such instability, we proposed a sign-sum statistic that counts the signs of the *t*-statistics for all possible subsets. The proposed method excludes genes affected by heterogeneity, thereby improving the reproducibility of gene ranking. We compared the sign-sum statistic with the *t*-statistic by a theoretical evaluation of the upper confidence limit. Through simulations and applications to real data sets, we show that the sign-sum statistic exhibits superior performance.

**Conclusion:**

We derive the sign-sum statistic for getting a robust gene ranking. The sign-sum statistic gives more reproducible ranking than the *t*-statistic. Using simulated data sets we show that the sign-sum statistic excludes hetero-type genes well. Also for the real data sets, the sign-sum statistic performs well in a viewpoint of ranking reproducibility.

**Electronic supplementary material:**

The online version of this article (doi:10.1186/s12920-016-0214-5) contains supplementary material, which is available to authorized users.

## Background

Detection of disease-associated markers plays a crucial role in gene screening for biological studies. In this field, statisticians seek to identify informative genes as candidates for further investigation. To this end, it is desirable to correctly rank genes according to their degree of differential expression. In such efforts, two-sample test statistics, such as the *t*-statistic and Wilcoxon sum-rank statistic, are widely used to rank genes based on gene expression data.

**Fig. 1:**
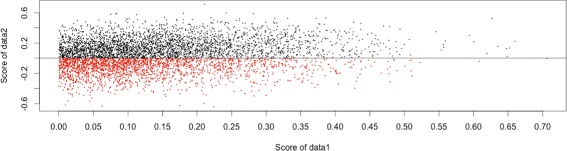
Scores for two independent data sets obtained using the *t*-statistic. The relative importance are evaluated based on the absolute values. *Red* points under the vertical line denotes the sign mismatched genes between the two data sets

However, the resultant gene rankings are often not reproducible among different data sets. Such irreproducibility may be caused by disease heterogeneity [1]. In fact, we can easily confirm this ranking irreproducibility in the microarray data used by [2] (see Application for more detail). That data set contains 51 non-metastatic samples and 46 metastatic samples from patients with breast cancer, a disease that is heterogeneous due to the existence of multiple subtypes [3]. We divided the full data into two independent sets (data1 and data2), transformed each data set such that the *t*-scores of data1 were positive without loss of generality, and then ranked genes using the *t*-statistic in data1 and data2 separately. Figure 1 shows the correspondence of these ranking scores. Some genes that were top-ranked in data1 had rather low scores in data2. Moreover, for many genes, even the signs of the *t*-scores were mismatched. This may be due to statistical variations caused by a sample size or heterogeneous factors in breast cancer. Thus, the simple *t*-statistic or correlation results in an unstable estimation that strongly depends on the dataset used.

Such heterogeneity was also discussed in a context of cancer outlier [4, 5] which developed the methods using the two-sample test statistic to detect genes that are over or down expressed in the subset of the disease group compared with the normal group. In this paper, we focus not on the subset but rather on the whole set. That is, our main aim is to detect the genes that are differentially expressed entirely in the disease group. Thus we develop the ranking method which has robustness for the heterogeneity.

Novel methods such as *lasso* are able to determine both highly ranked genes and classifiers simultaneously. However, [6] supported the importance of choosing a filtering method that yields a gene ranking corresponding to feature selection, rather than to the classification in machine-learning theory. In other words, pre-selection and evaluation of the resultant gene set must be separated from the classifier’s performance. Moreover, each top-ranked gene in itself must be informative or effective in some sense, e.g., robustness with respect to heterogeneity focused in this paper. Therefore we consider the ranking score independently derived for each gene, unlike [7], which took correlations between genes into consideration.

Because *t*-statistics and correlations strongly fluctuate due to sample variation, combining a sampling method with a two-sample test statistic should improve reproducibility. The effects of sampling method have been demonstrated by multiple studies, both theoretical [8] and applied [9]. Meanwhile, [10] presented counterarguments: several approaches to feature selection with ensemble learning by the sampling method are ineffective in terms of predictive ability, stability, and interpretability. Those authors concluded that the simple Student’s *t*-test exhibits superior performance in these regards. Dabney [11] also argued that the simple *t*-statistic is more accurate than the modified *t*-statistic and shrunken centroids. However, those authors’ conclusions are based on empirical studies in the context of particular data. We argue that the sampling method is effective from the standpoint of robustness with respect to heterogeneous factors; this is because heterogeneity represents a mixture of two or more classes, and we can easily imagine that sampling is the best way to capture heterogeneity to integrate information from many small subsample sets. To stabilize the performance of the simple *t*-statistic, we derived a sign-sum statistic that improves ranking reproducibility. This novel statistic repeatedly counts the sign of mean difference between subsets of the normal and disease groups. The sign-sum statistic is an extension of the Wilcoxon sum-rank statistic, which itself has superior robustness but an inferior power relative to the *t*-statistic [12]. We show the probabilistic result of the sign-sum statistic, and demonstrate its superior performance through simulations and applications to real data in this paper.

## Methods

### Derivation

Let *X*_*ij*_ be gene expression levels for samples *i*=1,⋯,*n*, genes *j*=1,…,*p*. We assume that all samples fall into either of two groups, 0 or 1, which denote normal and disease groups consisting of *n*_0_ and *n*_1_ samples respectively. Then, a two-sample *t*-statistic is defined by

1 $$\begin{array}{@{}rcl@{}} T_{j}={\frac{\sqrt{n}\left(\bar{X}_{j1}-\bar{X}_{j0}\right)}{s_{j}}}, \end{array} $$

where $\bar {X}_{jy}$ is the sample mean of group *y* for gene *j*. There are two options for *s*_*j*_, a pooled Student’s type or a non-pooled Welch’s type. In this paper, we use Welch’s *t*-statistic; therefore, *s*_*j*_ is written as $s_{j} = \sqrt {s_{1j}^{2} / \hat {\pi }_{1}+s_{0j}^{2}/ \hat {\pi }_{0}}$, where *s*_*yj*_ is the sample standard deviation of gene *j* and $\hat {\pi }_{y} = n_{y} / n$ for group *y*. Without loss of generality, we can assume that $\bar {X}_{j1}-\bar {X}_{j0}\geq 0$.

However, as discussed in Background, the *t*-statistics can fluctuate between two divided data sets, and even the signs of *t*-statistics can be mismatched. Therefore, we focus our attention to the signs of the *t*-statistics. If the *t*-statistics are evaluated by the full sample, the signs are positive over all genes by the assumption above. However, if the *t*-statistics are evaluated by subsets of the full sample, the signs may change, as shown in Fig. 1. Therefore, we derived a sign-sum statistic to count the signs of the *t*-statistics for all possible subsets.

We pick samples of sizes *a* and *b* from groups 1 and 0 respectively; thus, there are $\binom {n_{1}}{a}$ and $\binom {n_{0}}{b}$ combinations of subsets from each class. The sign-sum statistic is defined as

2 $$\begin{array}{@{}rcl@{}} {U^{S}_{j}} = \frac{1}{k_{0} k_{1}}{\sum\limits_{l=1}^{k_{1}}}{\sum\limits_{m=1}^{k_{0}}}~ \mathrm{H}(\bar{X}_{j1l}-\bar{X}_{j0m}), \end{array} $$

where $k_{1}=\binom {n_{1}}{a}, k_{0}=\binom {n_{0}}{b}, \mathrm {H}(x)$ is a Heaviside-step function that takes the value 0 if *x*<0 or 1 otherwise, and $\bar {X}_{jyt}$ is the sample mean of gene *j* in the *t*-th subset of group *y*. A larger value of the sign-sum statistic means that the signs of the *t*-statistics evaluated by subsamples are more stably positive. We can show that the sign-sum statistic is an extension of Wilcoxon’s sum-rank statistic; in fact, if *a* and *b* are equal to 1, then they are equivalent.

For comparison, we derived a *t*-statistic evaluated by subsamples. In a manner similar to the derivation of the sign-sum statistic, it is defined as

3 $$\begin{array}{@{}rcl@{}} {U^{T}_{j}}=\frac{1}{k_{0} k_{1}}{\sum\limits_{l=1}^{k_{1}}}{\sum\limits_{m=1}^{k_{0}}} \frac{\sqrt{a+b}~ (\bar{X}_{j1l}-\bar{X}_{j0m})}{s_{j}}. \end{array} $$

These statistics are described by the character *U* because they are members of *t*-statistics, as shown in Additional file 1. We compare the sign-sum statistic (2) with the *t*-statistic evaluated by subsamples (3) from the perspective of *t*-statistics in the next subsection.

By the assumption $\bar {X}_{j1}-\bar {X}_{j0}\geq 0$, the gene which has a larger score of the statistic is regarded as more informative for the detection of differentially expressed genes; thus, the gene ranking is obtained by sorting the values of the statistics in descending order over all genes.

### Robustness for heterogeneity

Heterogeneous disease factors can cause a mixture of two or more classes in some gene expression levels in the disease group. We call genes affected by such factors as “hetero genes”, and unaffected genes as “homo genes”. The sign-sum statistic can effectively detect such heterogeneity. To demonstrate this, here we provide a theorem about asymptotic confidence intervals.

Let *U* be a general two-sample *U*-statistic (we drop the gene index for simplicity). Because the *U*-statistic has the property of asymptotic normality, the asymptotic confidence interval is described as $\mathrm {E}[U] \pm (\sigma _{U} / \sqrt {n}) Z_{\alpha /2}$, where ${\sigma ^{2}_{U}}$ is an asymptotic variance of *U* and *Z*_*α*/2_ is the 100*α*/2 upper percentile of a standard normal distribution. Because *U*^*T*^ and *U*^*S*^ are members of *U*-statistics, these statistics are evaluated by the interval estimators as shown in Theorem 1.

#### **Theorem 1**

1. The asymptotic confidence interval of the *t*-statistic evaluated by subsamples with level *α* is
  4 $$\begin{array}{@{}rcl@{}} \frac{\sqrt{a+b} \ (\mu_{1}-\mu_{0})}{\sqrt{\cfrac{{\sigma_{1}^{2}}}{\pi_{1}}+\cfrac{{\sigma_{0}^{2}}}{\pi_{0}}}} \pm Z_{\alpha/2} \frac{(a+b)^{1/2}}{\sqrt{n}}  \end{array} $$
  if $\hat {\pi }_{1} \rightarrow \pi _{1}$ and $\hat {\pi }_{0} \rightarrow \pi _{0}$, where *π*_1_+*π*_0_=1 and *Z*_*α*/2_ is 100*α*/2 upper percentile of a standard normal distribution.
2. The asymptotic confidence interval of the sign-sum statistic (2) with level *α* is
  5 $$\begin{array}{@{}rcl@{}} \mathrm{E}[U^{S}] \pm Z_{\alpha/2} \frac{\tilde{\sigma}}{\sqrt{n}}, \end{array} $$
  if $\hat {\pi }_{1} \rightarrow \pi _{1}$ and $\hat {\pi }_{0} \rightarrow \pi _{0}$, where *Z*_*α*/2_ is 100*α*/2 upper percentile of a standard normal distribution, and
  6 $$\begin{array}{@{}rcl@{}} \mathrm{E}[U^{S}]&=&\mathrm{E}[G_{1}(V_{1})],  \end{array} $$
  7 $$\begin{array}{@{}rcl@{}} \tilde{\sigma}^{2} &=&\frac{a^{2}}{\pi_{1}}\text{Var} [G_{1} (V_{1})]+\frac{b^{2}}{\pi_{0}}\text{Var} [G_{0} (V_{0})], \end{array} $$
  where *G*_*y*_(*v*)=Pr(*W*_*y*_≤*v*) for *y*=0,1, and
  $$\begin{array}{@{}rcl@{}} V_{1} &=& \frac{1}{a}X_{11}, W_{1} =-\frac{1}{a}\sum\limits_{i=2}^{a} X_{1i} + \frac{1}{b} \sum\limits_{j=1}^{b} X_{0j}, \\ V_{0} &=& -\frac{1}{b}X_{01}, W_{0} =-\frac{1}{a}\sum\limits_{i=1}^{a} X_{1i} + \frac{1}{b} \sum\limits_{j=2}^{b} X_{0j}. \end{array} $$
  Here *X*_1_s and *X*_0_s are independently distributed with *F*_1_ and *F*_0_, which denote the distribution functions of gene expression levels of the disease and normal groups, respectively.

A proof of the Theorem 1 is given in Additional file 1. We note that *V*_1_−*W*_1_ and *V*_0_−*W*_0_ represent the mean differences in the disease and normal groups, respectively. A property of *U*-statistics allows us to evaluate the asymptotic variance of the sign-sum statistic by the conditional distribution of *W*_*y*_ given *V*_*y*_ for each group *y*. The difference between these two statistics is mainly due to the fact that the sign-sum statistic is the sum of the non-linear functions of the *t*-statistic evaluated by subsamples. As a result, information about *F*_1_ and *F*_0_ is strongly reflected in the sign-sum statistic as a result of changing *a* and *b*. We can discriminate hetero genes from homo genes by this property, as shown in the next subsection.

### The effects of different setting of parameters

Here we aim to remove heterogeneous factors by choosing *a* and *b* used in the sign-sum statistic, or equivalently controlling the subsample sizes from the disease and normal groups. If the *t*-value of a homo gene is larger than that of a hetero gene, then the *t*-statistic easily distinguishes the homo gene from the hetero gene. However, if the *t*-values of the two genes are equal, then the *t*-statistic will confuse these genes, because their confidence intervals are equal. Such confusing homo genes will be top-ranked by the sign-sum statistic if we find *a* and *b* such that

8 $$\begin{array}{@{}rcl@{}} U_{\text{homo}}^{T}=U_{\text{hetero}}^{T},~ U_{\text{homo}}^{S} > U_{\text{hetero}}^{S}. \end{array} $$

The difficulty and importance of considering such hetero genes is also discussed in [13] in the context of the false positive rate. The sign-sum statistic repeatedly counts the sign of the difference between the means of the disease and normal groups. Hence, the sign mismatches due to heterogeneity in the disease group would be effectively detected by a small *a* value, chosen such that the sample mean of the disease group fluctuates. This consideration is supported through numerical evaluations of specific situations, as described below.

Let all gene expression levels in the normal group follow *N*(0,1) without loss of generality. Then, the homo gene expression levels of the disease group follow $N(\mu _{1},{\sigma _{1}^{2}})$, and the hetero gene expression levels follow $\tau _{1} N(m_{1},{v_{1}^{2}})+ \tau _{2} N(m_{2},{v_{2}^{2}})$, where *τ*_1_ and *τ*_2_ are mixing proportions with *τ*_1_+*τ*_2_=1. Moreover, we constrain expectations of the *t*-statistics of these genes by equality in the limiting sense of probability convergence. That is,

9 $$\begin{array}{@{}rcl@{}} \frac{\mu_{1}-\mu_{0}}{\sqrt{\frac{{\sigma_{1}^{2}}}{\pi_{1}}+\frac{{\sigma_{0}^{2}}}{\pi_{0}}}} =\frac{\mu_{1}^{*}-\mu_{0}}{\sqrt{\frac{\sigma_{1}^{*2}}{\pi_{1}}+\frac{{\sigma_{0}^{2}}}{\pi_{0}}}}, \end{array} $$

where $\pi _{1}, \pi _{0}, \mu _{1}^{*},$ and $\sigma _{1}^{*}$ are the sample ratio of the disease group, the sample ratio of the normal groups, the expected mean, and the expected standard deviation of the hetero gene. The ranking by *t*-statistics fluctuates because the interval estimators of the hetero and homo genes are almost overlapping.

**Fig. 2:**
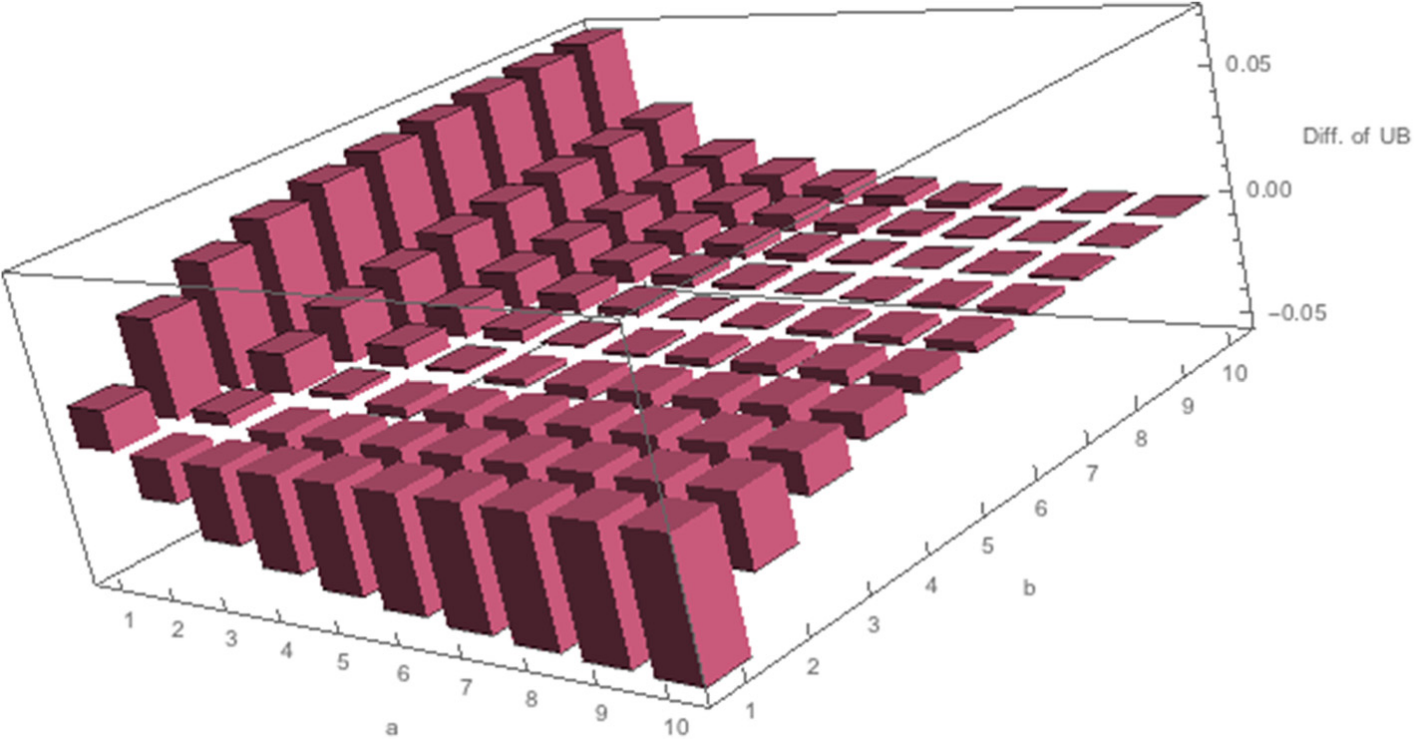
Difference in the 95 % upper confidence limits between homo and hetero genes. Each bounds is evaluated by (4) when $\pi _{1}=\pi _{0}=0.5, {\sigma _{1}^{2}}=1$, and *τ*_1_=*τ*_0_=0.5

The asymptotic confidence interval of the sign-sum statistic is not evaluated analytically because it has an integral form. In some situations, we can confirm that upper confidence limits of the sign-sum statistic differ between the homo and hetero genes. Figure 2 shows one such situation in the same setting as simulation (I) in Simulation. Thus, the sign-sum statistic can distinguish homo and hetero genes, whereas the *t*-statistic cannot. Figure 2 shows that (i) for each fixed value of *a* (sampling size from normal group), a larger value of *b* (sampling size from disease group) is better at discriminating these scenarios, and (ii) a smaller value of *a* tends to be superior. This is because disease heterogeneity affects the difference between a sensitive estimator of the mean in the disease group and a stable estimator of the mean in the normal group. Although it would be ideal to obtain an optimal setting for the sampling size in general situations, based on these observations we fixed *a* as 1 and allowed *b* to be 1,5, or 10 below. Below, the sign-sum statistic for each *a* and *b* is described as *s*_*a, b*_.

### Simulation

We carried out simple simulation studies to evaluate the performance of the sign-sum statistic. With the number of genes set at 1000, we generated expression levels for 100 homo genes and 100 hetero genes; the remaining 800 were non-informative genes whose expression level distributions were equal in the disease and normal groups. Gene expression levels in the normal group were assumed to be drawn from a standard normal distribution *N*(0,1) without loss of generality. Homo gene expression levels in the disease group were drawn from a normal distribution $N(1,{\sigma _{1}^{2}})$, and hetero gene expression levels in the disease group were drawn from a normal mixture distribution *τ*_1_*N*(0,1)+*τ*_2_*N*(*m*_2_,1), where *τ*_1_,*τ*_2_ are positive values with *τ*_1_+*τ*_2_=1. The mixture model suggests that a proportion *τ*_1_ of gene expression levels in the disease group cannot be discriminated from those in the normal group, as in real data.

We considered three situations in which the *t*-statistic confuses the homo and hetero genes by the constraint as (9) with different parameters: (I) ${\sigma _{1}^{2}}=1, \tau _{1}=0.5$, (II) $ {\sigma _{1}^{2}}=4, \tau _{1}=0.75$ and (III) ${\sigma _{1}^{2}}=1, \tau _{1}=0.25 $, with sample size *n*=200,1000 with equal *n*_0_ and *n*_1_. We compared the gene rankings among three statistics: simple *t*-statistic with subsamples, simple *t*-statistic without subsamples, and sign-sum statistic with 100 repetitions. The sampling sizes were fixed as *a*=1 and *b*=1 for the *t*-statistic, and as *a*=1 and *b*=1,5 and 10 for the sign-sum statistic. Robustness with respect to heterogeneity was calculated based on the number of homo genes in the top 100 ranking. Although ${U_{j}^{T}}$ and ${U_{j}^{S}}$ are defined by all possible subsets, in this case we only need to evaluate sufficient combinations to achieve convergence of the top 100 rankings as written in Additional file 2.

### Application

We compared the *t*-statistic with the sign-sum statistic using five real data sets [2, 14–17]. The data set in [2] (breast cancer data) contains 97 gene expression subjects for primary breast tumors in which 46 subjects are in relapsed group and 51 subjects are in relapse-free group for 5 years. We applied the same filtering used in [2], yielding a final full data set consisting of 97 samples and 5420 genes. The data set in [14] (cohort data) combine 454 gene expression samples from different diseases. We picked 32 samples from lung cancer tumors, 45 samples from pancreatic ductal adenocarcinoma tumors, and 70 samples from unaffected individuals, yielding a final full data set consisting of 147 samples and 863 genes. The data set in [15] (prostate cancer data) contains 6144 gene expressions for 455 prostate cancer tumors in which 103 subjects are determined as fusion status-positive and 352 subjects are determined as fusion status-negative. The data set in [16] (breast cancer data2) contains 17489 gene expressions for 286 breast cancer tumors in which 107 subjects are in relapsed group and 179 subjects are in relapse-free group within 5 years. The data set in [17] (leukemia data) contains 7129 gene expressions for 72 leukemia samples in which 47 subjects are in acute lymphoid leukemia group and 25 subjects are in acute myelogenous leukemia group.

For these data, the measure of reproducibility is given below. First, we divided the original data randomly into two data sets while maintaining the sample ratio of the disease and normal groups at the same value as in the full data set. After gene rankings were performed by the *t*-statistic and the sign-sum statistic, we selected the top-ranked 100 genes and counted the genes that overlapped between the two selections. This procedure was repeated for 100 trials, so we compared the *t*-statistic with the sign-sum statistic based on the mean and standard deviation of the overlapping counts. To account for the difference in sample sizes of the two datasets we also used *ORRS* (*OverlapRatio* to *RandomSelection*). For *p* genes, the *ORRS* for the top *k*-ranking is defined as

10 $$\begin{array}{@{}rcl@{}} \frac{1}{T}\sum\limits_{t=1}^{T}\frac{|S_{1t} \cap S_{2t}|}{N_{p,k}}, \end{array} $$

where $N_{p,k}=\sum _{i=0}^{k} i \binom {k}{i} \binom {p-k}{k-i}/\binom {p}{k}{=k^{2} / p}, S_{1t}$ and *S*_2*t*_ are the top *k*-ranked genes sets for two divided data on the *t*-th trial; in this case, *k*=100 and *T*=100. *N*_*p, k*_ refers to the expected overlap in gene number for a random selection. A larger *ORRS* value means that the selection is more reproducible than the random selection.

## Results and discussion

### The performance of the sign-sum statistic

**Fig. 3:**
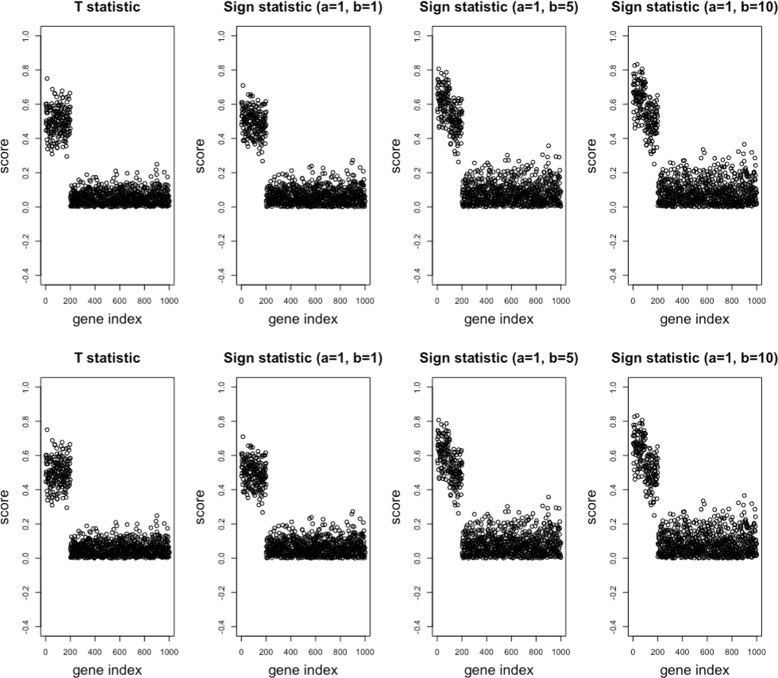
Scores obtained using the *t*-statistic, Wilcoxon sum-rank statistic, and sign-sum statistic with two sampling sizes. From *left* to *right*, *n*=200 (*upper*), *n*=1000 (*lower*). Horizontal axis denotes the gene indices. Vertical label denotes the ranking score. High score indicates relative importance for discrimination of class labels. The first 100 genes are homo informative genes, the next 100 are the hetero informative genes, and the last 800 genes are the non-informative genes

Table 1 shows the simulation results. We observe that the sign-sum statistic selected more homo genes highly associated with the class labels than the *t*-statistic. Overall, *s*_1,10_, the sign-sum statistic with sampling size 1 from the disease group and 10 from the normal group, performed the best in Situations (I) and (II). *s*_1,1_,*s*_1,5_,*s*_1,10_ were competitive and performed better than the *t*-statistic in Situation (III). These results confirmed the stable behavior of the sign-sum statistic, as shown in Fig. 2. Figure 3 also illustrates the superior performance of the sign-sum statistic, which shows one of the resulting ranking scores from the 100 trials. Homo and hetero genes were well discriminated by sign-sum statistic, but confused by the *t*-statistic. The ranking yielded better results in a large sample size (*n*=1000) than in a small sample size (*n*=200). When the sample size is 1000, almost all homo genes were ranked higher than hetero genes. Table 2 shows the application results, which indicated that the sign-sum statistic performed better with respect to these reproducibility measures. Overall, *s*_1,10_ performed the best, and this result corresponds to the simulation study, as shown in Table 1.

**Table 1:** The number of homo genes in the top 100 ranking: these are obtained using the *t* and sign-sum statistics. Means(sd) from 100 repetitions for each situations and sample size is written

*n*=200					
	*t*	*t*_1,1_	*s*_1,1_	*s*_1,5_	*s*_1,10_
Situation I	50.0 (4.12)	49.7 (3.67)	61.7 (3.20)	80.8 (2.56)	83.1 (2.36)
Situation II	49.9 (3.50)	49.3 (3.49)	48.9 (3.19)	56.5 (3.16)	57.8 (3.23)
Situation III	49.7 (3.09)	49.4 (3.31)	72.1 (2.77)	73.3 (3.12)	72.4 (3.14)
*n*=1000					
	*t*	*t*_1,1_	*s*_1,1_	*s*_1,5_	*s*_1,10_
Situation I	49.9 (4.19)	49.9 (4.08)	75.2 (2.98)	97.3 (1.20)	98.2 (1.00)
Situation II	50.1 (3.58)	49.9 (3.86)	47.6 (3.48)	64.0 (3.07)	67.2 (3.13)
Situation III	50.2 (3.65)	50.3 (3.82)	90.7 (2.19)	92.6 (2.04)	92.4 (1.98)

Two small subscripts for each statistic denote sampling sizes from the disease and normal groups in this order

**Table 2:** Reproducibility and ORRS: these values indicate mean(sd) and were evaluated by 100 random separations of the full data

*Reproducibility*	*t*	*t*_1,1_	*s*_1,1_	*s*_1,5_	*s*_1,10_
Breast cancer data	3.78 (1.92)	3.68 (1.99)	4.33 (2.13)	6.70 (2.77)	7.39 (3.37)
Cohort data	23.7 (4.94)	23.5 (4.86)	27.5 (5.70)	43.4 (5.39)	42.6 (5.28)
Prostate cancer data	33.4 (4.29)	32.6 (4.69)	39.6 (4.76)	31.4 (4.64)	29.4 (4.23)
Breast cancer data2	1.39 (1.35)	1.42 (1.31)	1.00 (1.10)	3.33 (1.80)	3.82 (1.91)
Leukemia data	32.3 (4.47)	31.8 (4.41)	34.2 (4.59)	37.4 (4.30)	37.1 (4.22)
*ORRS*	*t*	*t*_1,1_	*s*_1,1_	*s*_1,5_	*s*_1,10_
Breast cancer data	2.20 (1.11)	2.14 (1.16)	2.52 (1.24)	3.90 (1.62)	4.30 (1.96)
Cohort data	2.02 (0.42)	2.01 (0.42)	2.35 (0.49)	3.71 (0.46)	3.63 (0.45)
Prostate cancer data	20.0 (2.57)	19.5 (2.81)	23.7 (2.85)	18.8 (2.78)	17.6 (2.54)
Breast cancer data2	2.73 (2.64)	2.78 (2.57)	1.96 (2.16)	6.53 (3.53)	7.49 (3.75)
Leukemia data	19.3 (2.68)	19.1 (2.64)	20.5 (2.75)	22.4 (2.57)	22.2 (2.53)

### Discussion

Gene ranking procedures are not reproducible among different studies [18]. To obtain a robust ranking, ensemble or resampling methods are effective [8, 9]. Counterintuitively, however, resampling methods do not improve reproducibility [10]. In this paper, we evaluated a resampling method for robustness with respect to heterogeneity in a microarray study. We focused on the sign mismatch of *t*-scores in the context of a classification problem. We often found that the genes with large *t*-scores in the training data had small or sign-reversed *t*-scores in the test data. The sign-sum statistic was developed based on these two motivations. Using numerical simulation, we proved that the sign-sum statistic improves the robustness with respect to heterogeneity relative to the *t*-statistic. Furthermore, the sign-sum statistic allowed us to obtain a reproducible ranking in applications to real data. These conclusions were validated by an evaluation of the upper confidence limit (Theorem 1).

In the context of gene screening, *FDR* (*False Discovery Rate*) has been studied by novel methods such as *SAM* [19] and ranking procedure by *q*-values [20] for decisions about the cut-off value for gene ranking. It is less meaningful to focus on the cut-off value until we have a correct and stable gene ranking. Therefore, in this study, we focused on obtaining a reproducible gene ranking. Obtaining the cut-off value of the sign-sum statistic is a goal for future work.

In this paper, we focused on robustness with respect to heterogeneity. However, we should still confirm that resulting genes are informative. In fact, the cancer outlier methods, which focus on the hetero genes, provide high reproducibility. However, we consider that the top-ranked differentially expressesd genes in any rankings should be effective for the latter prediction problem. Although further study is needed for such discussion, we performed a simple examination to ensure a certain degree of predictive power. Table 3 shows thepredictive performance of DLDA (Diagonal Linear Discriminant Analysis) measured by AUC (Area Under the Curve). The AUC was calculated for all 100 trials used in Application, regarding randomly two divided datasets as training and test data. The scores were based on the top 10, 50 or 100 genes in every ranking. In particular, it shows that the *t*-statistic and sign-sum statistic have comparable predictive performance, although the DLDA predictor is constructed from each *t*-value for all genes. Thus the sign-sum statistic improves the ranking reproducibility without loss of predictive performance of the resultant genes.

**Table 3:** Test AUC for four real data sets: each predictor is constructed by the DLDA rule. These values indicate mean(sd) and were evaluated by 100 random separations of the full data

breast cancer data	AUC of the test data by DLDA				
	*t*	*t*_1,1_	*s*_1,1_	*s*_1,5_	*s*_1,10_
10 genes	0.698 (0.058)	0.698 (0.058)	0.698 (0.060)	0.684 (0.065)	0.679 (0.069)
50 genes	0.705 (0.046)	0.705 (0.047)	0.707 (0.050)	0.712 (0.051)	0.712 (0.050)
100 genes	0.711 (0.045)	0.710 (0.045)	0.712 (0.047)	0.718 (0.045)	0.718 (0.045)
Cohort data	AUC of the test data by DLDA				
	*t*	*t*_1,1_	*s*_1,1_	*s*_1,5_	*s*_1,10_
10 genes	0.744 (0.061)	0.743 (0.061)	0.751 (0.064)	0.755 (0.064)	0.771 (0.063)
50 genes	0.779 (0.057)	0.778 (0.057)	0.773 (0.053)	0.784 (0.053)	0.789 (0.054)
100 genes	0.782 (0.056)	0.781 (0.057)	0.778 (0.054)	0.781 (0.052)	0.784 (0.051)
Prostate cancer data	AUC of the test data by DLDA				
	*t*	*t*_1,1_	*s*_1,1_	*s*_1,5_	*s*_1,10_
10 genes	0.835 (0.025)	0.835 (0.026)	0.832 (0.025)	0.823 (0.027)	0.808 (0.032)
50 genes	0.846 (0.023)	0.845 (0.024)	0.847 (0.022)	0.836 (0.029)	0.829 (0.033)
100 genes	0.844 (0.024)	0.842 (0.025)	0.848 (0.021)	0.829 (0.030)	0.822 (0.033)
Breast cancer data2	AUC of the test data by DLDA				
	*t*	*t*_1,1_	*s*_1,1_	*s*_1,5_	*s*_1,10_
10 genes	0.612 (0.044)	0.614 (0.041)	0.611 (0.043)	0.595 (0.042)	0.581 (0.044)
50 genes	0.634 (0.040)	0.633 (0.040)	0.630 (0.042)	0.623 (0.039)	0.619 (0.040)
100 genes	0.637 (0.040)	0.636 (0.038)	0.636 (0.416)	0.630 (0.037)	0.626 (0.038)
Leukemia data	AUC of the test data by DLDA				
	*t*	*t*_1,1_	*s*_1,1_	*s*_1,5_	*s*_1,10_
10 genes	0.981 (0.017)	0.982 (0.017)	0.986 (0.014)	0.991 (0.012)	0.990 (0.016)
50 genes	0.992 (0.014)	0.992 (0.014)	0.988 (0.013)	0.994 (0.008)	0.994 (0.008)
100 genes	0.992 (0.016)	0.991 (0.017)	0.989 (0.013)	0.995 (0.006)	0.995 (0.009)

Gene ranking is an essential in biological investigations. In this study, we were motivated by the desire to identify robust and predictive biomarkers. Hetero genes may be informative for some patients, but uninformative in others. In this sense, hetero genes should be extracted from gene rankings if these predictive performance is eqaul to or less than that of homo genes.

## Conclusions

The *t*-statistic confuses homo and hetero genes as shown in the simulation study. The ranking irreproducibility would be caused by such heterogeneity also in the real data analysis. In fact, even the signs of *t*-statistics of many genes mismatch in the real data. We present the sign-sum statistic for getting robust ranking. Robustness for heterogeneity of the sign-sum statistic is shown by the evaluation of the upper confidence limit. We can get more reproducible ranking by the sign-sum statistic for simulated data which assumes that there are heterogeneous factors, for the breast cancer data which is known as the hetero disease and the data which includes different disease statuses.

## Availability of supporting data

The data sets supporting the results of this article are provided at the following database http://bioinformatics.nki.nl/data/van-t-Veer_Nature_2002/ and GEO under the accession number of GSE31568.

## Abbreviations

AUC, area under curve; DLDA, diagonal linear discriminant analysis

## Additional files

Additional file 1 Proof of theorem 1. We give the proof of theorem 1 in this file. (PDF 146 kb)

Additional file 2 R-code of the sign-sum statistic. We give an example of the R-code of the sign-sum statistic. (PDF 34.9 kb)
