# Supplementary material for: Reproducible detection of disease-associated markers from gene expression data
Source: BMC Med Genomics. 2016 Aug 18;9:53. doi: 10.1186/s12920-016-0214-5 (PMC4991096; doi:10.1186/s12920-016-0214-5)
Supplement: Additional file 1 — Proof of theorem 1. We give the proof of theorem 1 in this file. (PDF 146 kb) [file 12920_2016_214_MOESM1_ESM.pdf]

Additional file 1 : Proof of theorem 1

Definition 1 *Let X_{1i} and X_{0j} be random samples from groups 1 and 0, with $i = 1, 2, \cdots, n_1$ and $j = 1, 2, \cdots, n_0$ respectively. Then, a two-sample U-statistic is defined as follows:*

$$U = \frac{1}{\binom{n_1}{a}\binom{n_0}{b}} \sum \phi(X_{1i_1}, \cdots, X_{1i_a} : X_{0j_1}, \cdots, X_{0j_b}) \tag{1}$$

with the sum extending over all

$$1 \le i_1 < \cdots < i_a \le n_1; 1 \le j_1 < \cdots < j_b \le n_0, \tag{2}$$

where a and b are integers of n_1 and n_0 or smaller respectively, and ϕ is any real-valued function.

Proposition 1

1. *The t-statistic evaluated by subsamples (2.3) is a member of the two-sample U-statistics.*
2. *The sign-sum statistic (2.2) is a member of the two-sample U-statistics.*

Proof. We drop the gene index for simplicity.

1. We can rewrite (2.3) as

$$\begin{aligned} U^T &= \frac{1}{k_0 k_1} \sum_{l=1}^{k_1} \sum_{m=1}^{k_0} \sqrt{\frac{a+b}{n}} \frac{\bar{X}_{1l} - \bar{X}_{0m}}{s} \\ &= \frac{1}{\binom{n_1}{a}\binom{n_0}{b}} \sum \phi_T(X_{1i_i}, \cdots, X_{1i_a}; X_{0j_1}, \cdots, X_{0j_b}) \end{aligned}$$

   with the sum extending over all

$$1 \le i_1 < \cdots < i_a \le n_1; 1 \le j_1 < \cdots < j_b \le n_0, \tag{3}$$

   where

$$\phi_T(X_{1i_1}, \cdots, X_{1i_a}; X_{0j_1}, \cdots, X_{0j_b}) = \frac{\sqrt{a+b}\left(\frac{1}{a}\sum_{l=1}^{a} X_{1i_l} - \frac{1}{b}\sum_{m=1}^{b} X_{0j_m}\right)}{\sqrt{\frac{\sigma_1^2}{\pi_1} + \frac{\sigma_0^2}{\pi_0}}}. \tag{4}$$

2. We can rewrite (2.2) as

$$U^S = \frac{1}{\binom{n_1}{a}\binom{n_0}{b}} \sum \phi_S(X_{1i_1}, \cdots, X_{1i_a}; X_{0j_1}, \cdots, X_{0j_b}),$$

with the sum extending over all

$$1 \le i_1 < \cdots < i_a \le n_1; 1 \le j_1 < \cdots < j_b \le n_0,$$

where

$$\phi_S(X_{1i_1}, \cdots, X_{1i_a}; X_{0j_1}, \cdots, X_{0j_b}) = \phi_{\mathrm{S}}\left(\frac{1}{a}\sum_{l=1}^{a} X_{1i_l} - \frac{1}{b}\sum_{m=1}^{b} X_{0j_m}\right).$$

Lemma 1 *Let U be the two-sample U-statistic, that is*

$$U = \frac{1}{\binom{n_1}{a}\binom{n_0}{b}} \sum \phi(X_{1i_i}, \cdots, X_{1i_a} : X_{0j_1}, \cdots, X_{0j_b})$$

with the sum extending over all

$$1 \le i_1 < \cdots < i_a \le n_1; 1 \le j_1 < \cdots < j_b \le n_0.$$

Here the X_1's and X_0's are independently distributed according to F_1 and F_0, respectively.

(1)

$$\mathrm{Var}[U] = \sum_{i=1}^{a}\sum_{j=1}^{b} \frac{\binom{a}{i}\binom{n_1-a}{a-i}}{\binom{n_1}{a}} \frac{\binom{b}{j}\binom{n_0-b}{b-j}}{\binom{n_0}{b}} \sigma_{ij}^2,$$

where

$$\sigma_{ij}^2 = \mathrm{Cov}[\phi(X_{11}, \cdots, X_{1a}, X_{01}, \cdots, X_{0b}), \phi(X_{11}, \cdots, X_{1i}, X'_{1i+1}, \cdots, X'_{1a}, X_{01}, \cdots, X_{0j}, X'_{0j+1}, \cdots, X'_{0b})] \ge 0,$$

for any $i \in \{0, 1, \cdots, a\}, j \in \{0, 1, \cdots, b\}$. Here $X_1, X_1's$ and $X_0, X_0's$ are independently distributed according to F_1 and F_0 respectively.

(2) If

$$\begin{aligned}
\sigma_{10}^2 &= \mathrm{Cov}[\phi(X_{11}, X_{12}, \cdots, X_{1a}; X_{01}, X_{02}, \cdots, X_{0b}), \phi(X_{11}, X'_{12}, \cdots, X'_{1a}; X'_{01}, X'_{02}, \cdots, X'_{0b})] > 0,\\
\sigma_{01}^2 &= \mathrm{Cov}[\phi(X_{11}, X_{12}, \cdots, X_{1a}; X_{01}, X_{02}, \cdots, X_{0b}), \phi(X'_{11}, X'_{12}, \cdots, X'_{1a}; X_{01}, X'_{02}, \cdots, X'_{0b})] > 0,
\end{aligned}$$

$\sigma_{ab}^2 > 0$ and $\frac{n_1}{n_1+n_0} \to \pi_1$,then

$$\mathrm{Var}(\sqrt{n}U) \to \sigma_{av}^2 = \frac{a^2}{\pi_1}\sigma_{10}^2 + \frac{b^2}{1-\pi_1}\sigma_{01}^2.$$

Proof. See Lehmann (1999).

We consider the t-statistic evaluated by subsamples (2.3) and the sign-sum statistic (2.2). Now we drop subscript j for simplicity. Applying the central limit theorem for U-statistics, an upper confidence limit of these two statistics are evaluated as Theorem 2.1.

Theorem 2.1

1. *The asymptotic confidence interval of the t-statistic evaluated by subsamples (2.3) with level α is*

$$\frac{\sqrt{a+b}\ (\mu_1-\mu_0)}{\sqrt{\dfrac{\sigma_1^2}{\pi_1}+\dfrac{\sigma_0^2}{\pi_0}}} \pm Z_{\alpha/2}\left(\frac{a+b}{n}\right)^{1/2}, \tag{5}$$

if $\frac{n_1}{n} \to \pi_1$ and $\frac{n_0}{n} \to \pi_0$, where $\pi_1+\pi_0=1$.

2. *The asymptotic confidence interval of the sign-sum statistic (2.2) with level α is*

$$\mathrm{E}[U^S] \pm Z_{\alpha/2}\left(\frac{\tilde{\sigma}^2}{n}\right)^{1/2}, \tag{6}$$

where $Z_{\alpha/2}$ is $100\alpha/2$ upper percentile of the normal distribution and

$$\begin{aligned} \mathrm{E}[U^S] &= \mathrm{E}[G_1(V_1)], & (7)\\ \tilde{\sigma}^2 &= \frac{a^2}{\pi_1}\mathrm{Var}[G_1(V_1)] + \frac{b^2}{\pi_0}\mathrm{Var}[G_0(V_0)], & (8) \end{aligned}$$

where $G_y(x) = \Pr(W_y \le x)$ for $y=0,1$ and

$$\begin{aligned} V_1 &= \frac{1}{a}X_{11}, W_1 = -\frac{1}{a}\sum_{i=2}^{a} X_{1i} + \frac{1}{b}\sum_{j=1}^{b} X_{0j},\\ V_0 &= -\frac{1}{b}X_{01}, W_0 = -\frac{1}{a}\sum_{i=1}^{a} X_{1i} + \frac{1}{b}\sum_{j=2}^{b} X_{0j}, \end{aligned}$$

here X_1s and X_0s are independently distributed according to F_1 and F_0, which denote the distribution function of gene expression levels of the disease and normal groups respectively.

Proof.

1. By applying Theorem 1, we find that

$$\mathrm{Var}(\sqrt{n}U^S) \to \sigma^2_{\mathrm{AVT}} = \frac{a^2}{\pi_1}\sigma^2_{10} + \frac{b^2}{\pi_0}\sigma^2_{01},$$

where

$$\begin{aligned}
\sigma^2_{10} &= \mathrm{Cov}[\phi_T(X_{11}, X_{12}, \cdots, X_{1a}; X_{01}, X_{02}, \cdots, X_{0b}), \phi_T(X_{11}, X'_{12}, \cdots, X'_{1a}; X'_{01}, X'_{02}, \cdots, X'_{0b})] \\
&= \frac{a+b}{\frac{\sigma_1^2}{\pi_1} + \frac{\sigma_0^2}{\pi_0}} \mathrm{Cov}\left[\frac{1}{a}(X_{11} + \sum_{i=2}^{a} X_{1i}) - \frac{1}{b}\sum_{j=1}^{b} X_{0j}, \frac{1}{a}(X_{11} + \sum_{i=2}^{a} X'_{1i}) - \frac{1}{b}\sum_{j=1}^{b} X'_{0j}\right] \\
&= \frac{a+b}{\frac{\sigma_1^2}{\pi_1} + \frac{\sigma_0^2}{\pi_0}} \mathrm{Cov}\left[\frac{1}{a}X_{11}, \frac{1}{a}X_{11}\right] \\
&= \frac{a+b}{\frac{\sigma_1^2}{\pi_1} + \frac{\sigma_0^2}{\pi_0}} \frac{\sigma_1^2}{a^2}
\end{aligned}$$

and

$$\sigma^2_{01} = \frac{a+b}{\frac{\sigma_1^2}{\pi_1} + \frac{\sigma_0^2}{\pi_0}} \frac{\sigma_0^2}{b^2}$$

because of the independence of X_1's and X_0's. Therefore we can prove that

$$\begin{aligned}
\sigma^2_{\mathrm{AVT}} &= \frac{a^2}{\pi_1}\frac{a+b}{\frac{\sigma_1^2}{\pi_1} + \frac{\sigma_0^2}{\pi_0}}\frac{\sigma_1^2}{a^2} + \frac{b^2}{\pi_0}\frac{a+b}{\frac{\sigma_1^2}{\pi_1} + \frac{\sigma_0^2}{\pi_0}}\frac{\sigma_0^2}{b^2} \\
&= a + b.
\end{aligned}$$

2. By applying Proposition 1 and Lemma 1, we find that the asymptotic variance of U^S is

$$\sigma^2_{\mathrm{AVS}} = \frac{a^2}{\pi_1}\tilde{\sigma}^2_{10} + \frac{b^2}{\pi_0}\tilde{\sigma}^2_{01},$$

where

$$\begin{aligned}
\tilde{\sigma}^2_{10} &= \mathrm{Cov}[\phi_{\mathrm{S}}(X_{11}, X_{12}, \cdots, X_{1a}; X_{01}, X_{02}, \cdots, X_{0b}), \phi_{\mathrm{S}}(X_{11}, X'_{12}, \cdots, X'_{1a}; X'_{01}, X'_{02}, \cdots, X'_{0b})] \\
&= \mathrm{Cov}[\phi_{\mathrm{S}}(\frac{1}{a}\sum_{i=1}^{a} X_{1i} - \frac{1}{b}\sum_{j=1}^{b} X_{0j}), \phi_{\mathrm{S}}(\frac{1}{a}(X_{11} + \sum_{i=2}^{a} X'_{1i}) - \frac{1}{b}\sum_{j=1}^{b} X'_{0j})] \\
&= \mathrm{Cov}[\phi_{\mathrm{S}}(\frac{1}{a}X_{11} - (\frac{1}{b}\sum_{j=1}^{b} X_{0j} - \frac{1}{a}\sum_{i=2}^{a} X_{1i})), \phi_{\mathrm{S}}(\frac{1}{a}X_{11} - (\frac{1}{b}\sum_{j=1}^{b} X'_{0j} - \frac{1}{a}\sum_{i=2}^{a} X'_{1i}))] \\
&= \mathrm{Cov}[\phi_{\mathrm{S}}(U_1 - V_1), \phi_{\mathrm{S}}(U_1 - V'_1)] \\
&= \mathrm{E}[\phi_{\mathrm{S}}(U_1 - V_1)\phi_{\mathrm{S}}(U_1 - V'_1)] - \mathrm{E}[\phi_{\mathrm{S}}(U_1 - V_1)]\mathrm{E}[\phi_{\mathrm{S}}(U_1 - V'_1)],
\end{aligned}$$

where

$$U_1 = \frac{1}{a} X_{11}, V_1 = \frac{1}{b} \sum_{j=1}^{b} X_{0j} - \frac{1}{a} \sum_{i=2}^{a} X_{1i}, V_1^{'} = \frac{1}{b} \sum_{j=1}^{b} X_{0j}^{'} - \frac{1}{a} \sum_{i=2}^{a} X_{1i}^{'}$$

are all independent.

$$\begin{aligned}
\mathrm{E}[\hat{S}] &= \mathrm{E}[\phi_{\mathrm{S}}(U_1 - V_1)] \\
&= \mathrm{E}[\phi_{\mathrm{S}}(U_1 - V_1^{'})] \\
&= \mathrm{E}[\mathrm{E}[\phi_{\mathrm{S}}(U_1 - V_1)|U_1]] \\
&= \mathrm{E}[\mathrm{P}(V_1 \leq U_1|U_1)] \\
&= \mathrm{E}[\int_{-\infty}^{U_1} f_{V_1|U_1}(v_1) dv_1] \\
&= \mathrm{E}[\int_{-\infty}^{U_1} f_{V_1}(v_1) dv_1] \\
&= \mathrm{E}[G_1(U_1)]. \qquad (9)
\end{aligned}$$

$$\begin{aligned}
\mathrm{E}[\phi_{\mathrm{S}}(U_1 - V_1)\phi_{\mathrm{S}}(U_1 - V_1^{'})] &= \mathrm{E}[\mathrm{E}[\phi_{\mathrm{S}}(U_1 - V_1)\phi_{\mathrm{S}}(U_1 - V_1^{'})|U_1]] \\
&= \mathrm{E}[\mathrm{E}[\phi_{\mathrm{S}}(U_1 - V_1)|U_1]\mathrm{E}[\phi_{\mathrm{S}}(U_1 - V_1^{'})|U_1]] \\
&= \mathrm{E}[G_1(U_1)^2].
\end{aligned}$$

Therefore

$$\tilde{\sigma}_{10}^2 = \mathrm{Var}[G_1(U_1)]$$

and similarly

$$\tilde{\sigma}_{01}^2 = \mathrm{Var}[G_0(U_0)],$$

where

$$U_0 = -\frac{1}{b} X_{01}, V_0 = -\frac{1}{a} \sum_{i=1}^{a} X_{1i} + \frac{1}{b} \sum_{j=2}^{b} X_{0j}.$$

Consequently we can show that

$$\sigma_{\mathrm{AVS}}^2 = \frac{a^2}{\pi_1} \mathrm{Var}[G_1(U_1)] + \frac{b^2}{\pi_0} \mathrm{Var}[G_0(U_0)]. \qquad (10)$$

References

[1] Lehmann, E, L. (1999). *Elements of large-sample theory*, Springer.
