# Supplementary material for: Reproducible detection of disease-associated markers from gene expression data
Source: BMC Med Genomics. 2016 Aug 18;9:53. doi: 10.1186/s12920-016-0214-5 (PMC4991096; doi:10.1186/s12920-016-0214-5)
Supplement: Additional file 2 — R-code of the sign-sum statistic. We give an example of the R-code of the sign-sum statistic. (PDF 34.9 kb) [file 12920_2016_214_MOESM2_ESM.pdf]

Additional file 2 : R-code of the sign-sum statistic

```
rpranking=function(data,lab,a,b,rank.num,conv.time){
  #inputs
    #data : data matrix
    #lab : indicator vector of clinical statuses(1 or 0)
    #a :  sampling number from lab 1 (range : from 1 to n1)
    #b :  sampling number from lab 0 (range : from 1 to n0)
    #rank.num : ranking number you want to get (range : from 1 to p)
    #conv.time : scale for convergence (range : 1 and over)

  #outputs
    #ranking : resulting ranking
    #sign.score : value of the sign-sum statistics
    #steps : discrete time until convergenece


n = dim(data)[1] ; n1 = length(which(lab==1)) ; n0 = n-n1
p = dim(data)[2]
pi1 = n1/n ; pi0 = 1-pi1
num1 = which(lab==1)
num0 = which(lab==0)


sign.score = numeric(p)
k = 1
time = 0
selection = numeric(p)


  while((time < conv.time) || (length(which(matrix(sign.score[order(-sign.score)[1:rank.num]],
  rank.num,rank.num)-t(matrix(sign.score[order(-sign.score)[1:rank.num]],
  rank.num,rank.num))==0)) != rank.num)){

  s1 = sample(num1,a)
  s0 = sample(num0,b)

  if(a!=1){
```

```
    u1 = apply(data[s1,],2,mean)
    } else{
    u1 = data[s1,]
    }

  if(b!=1){
    u0 = apply(data[s0,],2,mean)
    } else{
    u0 = data[s0,]
    }

  score = u1-u0
  sign.score = sign.score+sign(score)
  selection.pre = selection
  selection = order(-abs(sign.score))[1:rank.num]

  if((setequal(selection,selection.pre)^1)==1){
     time = time+1
  } else{
  time = 0
  }
    k = k+1
  }

score = sign.score / k
selection = order(-abs(score))[1:rank.num]


return(list(ranking=selection,sign.score=abs(score),steps=k))
}
```
